# Where Is the Lactate Coming From? An Unusual Presentation of Persistent Lactic Acidosis

**DOI:** 10.7759/cureus.104328

**Published:** 2026-02-26

**Authors:** Waleed Sadiq, Madeeha Subhan Waleed

**Affiliations:** 1 Pulmonary and Critical Care Medicine, Robert Wood Johnson (RWJ) Barnabas Health, New Jersey, USA; 2 Internal Medicine, Lower Bucks Hospital, Bristol, USA

**Keywords:** acidosis lactic, colorectal cancer, critical care, : metformin, metformin induced lactic acidosis

## Abstract

Persistent lactic acidosis in patients with metastatic colorectal cancer is uncommon and often attributed to impaired hepatic clearance from liver metastases. Acute worsening, however, may signal reversible metabolic derangements, including medication-related toxicity.

A 64-year-old male with type 2 diabetes mellitus, hypertension, and metastatic colorectal cancer to the liver presented with weakness, lethargy, and inability to tolerate oral intake for three days. His baseline lactate was persistently elevated (5-7 mmol/L) over six months. On presentation, he was hypotensive [blood pressure (BP) 82/41 mmHg], tachycardic [heart rate (HR) 122 bpm], febrile (101°F), and drowsy. Labs showed acute kidney injury (AKI) (Cr 2.7 mg/dL), hyperkalemia [Potassium (K) 6.1 mmol/L], severe metabolic acidosis (bicarbonate 4 mmol/L), transaminitis [aspartate aminotransferase (AST) 424, alanine transaminase (ALT) 576], and lactate 18 mmol/L. Complete blood count showed a white blood cell (WBC) count of 19,000/µL. Computed tomography (CT) of the abdomen revealed the known 7 cm colorectal mass with multiple hepatic metastases, moderate ascites, and no obstruction or ischemia. Despite aggressive intravenous (IV) fluids and vasopressors, lactate rose to 20 mmol/L, and urine output remained negligible.

Medication review revealed metformin use, raising suspicion for metformin-associated lactic acidosis (MALA) in the setting of AKI. Nephrology consultation was obtained, and continuous renal replacement therapy (CRRT) was initiated. Lactate declined to 12 mmol/L at four hours and 6 mmol/L at 12 hours. Hemodynamics improved, vasopressors were discontinued, urine output increased, and creatinine and bicarbonate normalized on day two. Eventually, the patient was successfully extubated, tolerated oral intake, and was discharged home after completing antibiotics.

This case illustrates multifactorial lactic acidosis: baseline elevation from liver metastases, superimposed type A lactic acidosis from sepsis, and type B lactic acidosis from metformin accumulation. Early recognition and initiation of CRRT were critical for rapid lactate clearance and clinical recovery.

In patients with baseline lactic acidosis due to metastatic liver disease, sudden lactate spikes should prompt evaluation for reversible causes, including renal dysfunction and medication toxicity. Multidisciplinary management and renal support can be lifesaving.

## Introduction

Lactic acidosis is defined biochemically by elevated arterial or venous lactate levels, typically >4 mmol/L, alongside acidemia (pH<7.35). It frequently complicates shock states, severe infections, and metabolic derangements, and is strongly associated with adverse outcomes in hospitalized patients. Historically viewed predominantly as a marker of tissue hypoxia, lactate is now recognized as an active intermediate in cellular metabolism with diverse roles beyond anaerobic glycolysis.

Lactate is produced from pyruvate via lactate dehydrogenase (LDH), principally during glycolysis, and serves to regenerate nicotinamide adenine dinucleotide (NAD⁺), allowing glycolysis to continue when pyruvate oxidation is limited. Contrary to earlier dogma, lactate is not merely a metabolic waste product; it functions as a key metabolic intermediate and substrate, shuttled between tissues such as muscle, liver, and brain for oxidation or gluconeogenesis. This “lactate shuttle” concept underscores the dynamic role of lactate in metabolic networks and energy homeostasis. Elevated lactate reflects enhanced production, impaired clearance, or both, with major clearance occurring via hepatic and renal pathways [1].

It is broadly categorized into type A, due to tissue hypoperfusion/hypoxia, and type B, arising from conditions such as liver disease, malignancy, or toxins/medications. Type B lactic acidosis is an uncommon but recognized complication in patients with extensive liver metastases, where impaired hepatic lactate clearance and tumor‑associated metabolic dysregulation contribute to elevated lactate levels. Case reports describe severe type B lactic acidosis in solid tumors, including colorectal cancer with liver involvement [2].

Clinical presentation of Lactic acidosis is heterogeneous with symptoms reflective of the underlying condition and can include tachypnea, hypotension, altered mental status, shock, and or severe acidemia exhibiting as arrhythmias. Diagnostic approach involves obtaining arterial blood gas, confirming acidosis along withan elevated anion gap, serial lactate measurement, and other pertinent imaging.

Metformin, a widely used antidiabetic, is renally excreted and may accumulate in patients with acute kidney injury (AKI) or renal dysfunction. While the overall risk of lactic acidosis with metformin is low, metformin-associated lactic acidosis (MALA) is a serious and potentially fatal complication when it occurs, especially in the presence of precipitating factors such as sepsis, hypoperfusion, or organ failure. The mechanism described for metformin-induced lactic acidosis, or MALA, is inhibition of hepatic gluconeogenesis, a major pathway for lactate clearance [3, 4].

MALA is associated with high mortality, with a range between 30%-50% [2]. So MALA must be recognized early and treated as soon as possible. Keeping this background information in mind, we present a case of MALA in a patient with persistent lactic acidosis due to hepatic metastasis leading to impaired clearance of lactate. Making it challenging to diagnose this medical condition.

## Case presentation

A 64‑year‑old male with a history of type 2 diabetes mellitus, hypertension, and metastatic colorectal cancer with liver metastases presented to the emergency department complaining of weakness, lethargy, and inability to tolerate oral intake for three days. He had a 30-pack-year smoking history and occasional alcohol use, and he previously worked as a plumber. Six months earlier, he had been diagnosed with colorectal cancer after presenting with weight loss, abdominal pain, and jaundice; computed tomography (CT) imaging revealed a mass with liver metastases, and a colonoscopy with biopsy confirmed malignancy. He was undergoing routine surveillance with positron emission tomography (PET) scans and was treated with fluorouracil, leucovorin, and oxaliplatin (FOLFOX)‑based chemotherapy.

Over the past six months, he had multiple admissions for spontaneous bacterial peritonitis, requiring paracentesis and intravenous (IV) antibiotics despite outpatient prophylactic antibiotics. Baseline lactic acid levels had been persistently elevated at 5-7 mmol/L, attributed to metastatic liver disease. On presentation, the patient was lethargic, jaundiced, confused, and dehydrated. He exhibited generalized abdominal tenderness. Vital signs are shown in Table 1.

**Table 1:** Vital signs at presentation

Parameter	Patient Value	Normal Adult Range
Systolic Blood Pressure (SBP)	82 mmHg	90–120 mmHg
Diastolic Blood Pressure (DBP)	41 mmHg	60–80 mmHg
Mean Arterial Pressure (MAP)	~55 mmHg	70–100 mmHg (≥65 mmHg minimum target in shock)
Heart Rate	122 bpm	60–100 bpm
Respiratory Rate	27 breaths/min	12–20 breaths/min
Temperature	101°F (38.3°C)	36.1–37.2°C (97–99°F)

Laboratory findings are described in Table 2. 

**Table 2:** Laboratory findings at admission

Parameter	Patient Value	Normal Adult Reference Range
White Blood Cell (WBC) Count	19,000/µL	4,000–11,000/µL
Creatinine	2.7 mg/dL (baseline 1.7 mg/dL)	0.6–1.3 mg/dL
Potassium (K⁺)	6.1 mmol/L	3.5–5.0 mmol/L
Bicarbonate (HCO₃⁻)	4 mmol/L	22–28 mmol/L
Aspartate Aminotransferase (AST)	424 U/L	10–40 U/L
Alanine Aminotransferase (ALT)	576 U/L	7–56 U/L
Alkaline Phosphatase (ALP)	380 U/L	44–147 U/L
Lactate	18 mmol/L	0.5–2.0 mmol/L

Imaging was performed in the emergency room (ER). CT abdomen demonstrated the known 7 cm colorectal mass with multiple hepatic metastases and moderate ascites without evidence of bowel obstruction or ischemia.

The patient was treated for sepsis, receiving 30 mL/kg IV fluids and broad‑spectrum antibiotics (meropenem). He became increasingly drowsy, necessitating intubation for airway protection after discussion with family regarding full resuscitation status. Despite resuscitation, repeat lactate increased to 20 mmol/L within two hours. Clinically, he developed worsening hypotension [blood pressure (BP) 79/40 mmHg] and tachycardia [heart rate (HR) 140 bpm], requiring norepinephrine and vasopressin infusions via a right internal jugular central line. Urine output remained minimal, consistent with anuria despite fluid resuscitation.

Medication review revealed outpatient use of metformin, along with amlodipine, furosemide, and spironolactone - all held on admission. Given the presence of AKI and worsening metabolic acidosis, MALA was suspected. Nephrology was consulted, and a temporary hemodialysis catheter was placed. The patient was initiated on continuous renal replacement therapy (CRRT).

Lactate levels trended downward after CRRT initiation. At four hours, lactate was 12 mmol/L and 6 mmol/L at 12 hours. Within 24 hours, his hemodynamic status improved, vasopressors were discontinued, urine output increased to 20 mL/hr, bicarbonate improved to 19 mmol/L, and creatinine declined to 1.5 mg/dL. Antibiotics and supportive care were continued for presumed sepsis. The patient was extubated successfully and passed a speech therapy evaluation for oral intake.

By day three, he was transferred from the intensive care unit (ICU) to the general medicine floor, completed his antibiotic course, and CRRT access was removed. He was discharged home in stable condition with follow‑up arrangements.

## Discussion

This case illustrates the complex, multifactorial nature of lactic acidosis in a patient with advanced malignancy and multiple comorbidities. Several overlapping mechanisms likely contributed to the elevated lactate levels. Impaired lactate clearance due to extensive liver metastases can significantly reduce hepatic metabolism of lactate, leading to chronic low-grade lactic acidosis. Although type B lactic acidosis in solid tumors is rare, it has been documented, particularly in patients with liver involvement [2].

Sepsis and hypoperfusion further exacerbate lactate accumulation. Systemic infection and hypotension increase anaerobic metabolism, contributing to type A lactic acidosis. These conditions act synergistically with impaired hepatic clearance to worsen metabolic derangements.

MALA is another potential contributor. Although MALA is rare, accumulation of metformin in the setting of acute kidney injury can impair pyruvate metabolism and gluconeogenesis, increasing lactate production and reducing clearance. Clinical recognition is crucial, as MALA can be life-threatening. Chang et al. emphasized that hemodialysis is the treatment of choice and should be initiated urgently to prevent serious complications. MALA should be suspected in patients presenting with a wide anion gap metabolic acidosis and elevated blood lactate, even in non-diabetic individuals on metformin [5]. Early intervention can be life-saving. Yusim et al. described the early use of hemodialysis to correct acidosis and recommended adjunctive therapy with tris-hydroxymethyl aminomethane (THAM) buffer alongside dialysis [6]. The incidence of MALA is estimated at approximately three to nine cases per 100,000 patient-years [7], with impaired renal function being the primary risk factor for metformin accumulation [8]. 

 Recognition of MALA is challenging because metformin rarely causes lactic acidosis in isolation; most cases occur in the presence of precipitating factors such as renal, hepatic, or hemodynamic compromise [3]. Evidence suggests that metformin is generally safe in patients with mild-to-moderate renal impairment, provided contraindications are respected.

Finally, renal replacement therapy plays a critical role in severe cases. CRRT facilitates rapid lactate clearance and correction of acid-base disturbances, allowing time for recovery of kidney function and stabilization of hemodynamics. This case underscores the importance of careful medication review, early renal support, and multidisciplinary management in critically ill patients with elevated lactate.

## Conclusions

In patients with baseline lactic acidosis due to metastatic liver disease, sudden worsening of lactic acid levels should alert clinicians to reversible contributors such as renal impairment, sepsis, and drug‑induced toxicity. Early recognition and prompt initiation of renal replacement therapy can lead to rapid clinical stabilization and improved outcomes.
